# Inhibitory control and stress biomarkers in response to social media stimuli: a Go/No-Go study using heart rate variability, alpha-amylase and oxytocin

**DOI:** 10.3389/fpsyt.2026.1872039

**Published:** 2026-08-19

**Authors:** Myriam Carbonell-Colomer, Elena Bernabéu-Brotóns, Carlos Marchena-Giráldez, Jose Joaquin Ceron, Silvia Martínez-Subiela, María Botía-González

**Affiliations:** 1 Faculty of Education and Psychology, Universidad Francisco de Vitoria, Pozuelo de Alarcón, Madrid, Spain; 2 Interlab-UMU, Interdisciplinary Laboratory of Clinical Analysis, Regional Campus of International Excellence ‘Campus Mare Nostrum’, University of Murcia, Murcia, Spain

**Keywords:** heart rate variability (SDNN), inhibitory control, oxytocin, salivary alpha-amylase, social media, stress, user profiles

## Abstract

Problematic social media use is increasingly recognized as a heterogeneous phenomenon; yet the cognitive and psychophysiological mechanisms underlying distinct user profiles remain poorly understood. This study examines the relationship between cognitive control, stress regulation, and the psychophysiological response to social media stimuli using a multidimensional approach. Using a three-phase protocol (baseline, task, and recovery), inhibitory control was assessed alongside objective biomarkers—heart rate variability (SDNN), salivary alpha-amylase (sAA), and salivary oxytocin—across three distinct psychophysiological profiles. The functional profile (FP) showed the highest cognitive performance (M = 76.75, SD = 2.61), a significant within-profile post-task SDNN increase (p = .003), and a progressive decrease in salivary sAA from baseline to the recovery phase (p = .001). The compulsive use profile (CUP) exhibited intermediate performance (M = 73.44) with a descriptive pattern of sympathetic reactivity during exposure and apparent subsequent autonomic recovery (p = .033, not surviving Bonferroni correction); this pattern is considered exploratory and should be interpreted with caution. The distress-associated profile (DAP) showed significantly more omissions both in the general task (H (2) = 6.47, p = .039) and specifically in response to social media stimuli (H (2) = 6.36, p = .042), as well as significantly fewer correct rejections to social media stimuli (H (2) = 6.36, p = .042), compared to the functional profile (DAP vs. FP: U = 69.00, Z = -2.55, p = .011). No significant between-group differences were found in false alarms or reaction time to social media stimuli. Taken together, these preliminary findings suggest that problematic social media use may not be homogeneous: while some users maintain recovery mechanisms, others show patterns suggestive of heightened reactivity and reduced biological flexibility, suggesting that, if replicated in larger and more controlled samples, these findings may inform the development of profile-differentiated intervention approaches.

## Introduction

Problematic social media use is increasingly recognized as a heterogeneous phenomenon, with accumulating evidence indicating that not all users who engage extensively with social media experience the same psychological or physiological consequences. Understanding this heterogeneity requires moving beyond behavioral self-report measures toward an integrated, multidimensional assessment that combines cognitive performance with objective physiological indicators. The present study addresses this need by examining inhibitory control alongside multiple stress biomarkers across distinct user profiles, with the aim of determining whether these profiles are psychophysiologically distinguishable.

Despite their recent emergence, social media platforms have established themselves as a widespread social phenomenon. Their ease of access, wide range of options and user-friendliness enable users to remain connected, entertained and informed, which has led to a large and ever-growing user base.

Given this exponential growth, it is unsurprising that scholarly attention to its potential problematic use, operationalized in this study as Problematic Smartphone and Social media Use (PSNUS), has grown substantially (1, 2), as well as its link to psychological distress (anxiety or depression) (3, 4), physical distress (such as insomnia and a sedentary lifestyle) (5) and even its potential addictive nature (6).

The constant presence of social media notifications in modern life acts as a socially relevant stimulus, capable of activating attentional, motivational and social systems (7) and thus providing reinforcing value for all users (8, p.14). However, this response does not appear to manifest uniformly across all users (9), in turn with heterogeneous consequences across individuals.

The present study is primarily framed within the stress-strain-outcome (SSO) model (10, 11), which posits that individual predisposing factors shape emotional strain in response to environmental stressors, leading to differentiated behavioral and physiological outcomes. This framework is complemented by considerations from the SORCAN model (12) and the I-PACE model (13) to interpret profile-specific patterns of emotional regulation and compensatory use, respectively. Rather than seeking to resolve the debate between addiction-based and distress-based conceptualizations of problematic social media use, this study adopts a profile-based approach, examining whether distinct psychophysiological signatures, rather than a single unifying mechanism, characterize different patterns of social media engagement.

Problems associated with social media use have been linked to symptoms similar to those described in substance use disorders (14). Due to the ‘confirmatory approach’, which involves reusing the criteria for substance use disorder to define and assess behavioral addictions (6), the vast majority of these studies have examined the core characteristics of substance addiction; some of these characteristics include attention, impulsivity, inhibitory control, and emotional distress associated with consumption or abstinence (15–17).

This approach has found significant scientific relevance in the consequences and characteristics of this more impulsive/problematic or addictive profile using similar techniques; for example, the use of the Go/Nogo paradigm, the gold standard for measuring the ability to suppress irrelevant stimuli (inhibitory control), impulsivity and attention. These core executive functions have been shown to be compromised in individuals with substance and behavioral addictions (18–21).

The theoretical rationale for applying the Go/NoGo paradigm to the study of problematic social media use stems from the dual-process model of addictive behavior, which posits that addictive and compulsive behaviors are maintained by an imbalance between automatic, reward-driven approach tendencies toward salient cues and the top-down inhibitory control needed to suppress these tendencies (22, 23). Within this framework, social media notifications function as conditioned cues that, through repeated association with social and emotional reward, acquire incentive salience and trigger prepotent approach responses. The Go/NoGo paradigm is therefore particularly well suited to capture the behavioral signature of this imbalance, as it directly measures the capacity to suppress a prepotent response (Go) when confronted with a salient but currently irrelevant cue (NoGo). Impaired performance on this task, manifested as increased omissions, false alarms, or reduced discriminability, would therefore be expected to reflect a reduced capacity to regulate automatic responses to socially salient digital cues, providing a behavioral correlate of the broader self-regulatory difficulties theorized in models of problematic social media use.

Impulsivity is regarded as a predictor of addiction (24), as well as a personality trait closely linked to the risk of problematic use and self-regulation of such use (25). Research has demonstrated a direct link between impaired inhibitory control and a greater attentional bias towards addiction-related stimuli (26, 27); for example, alcoholics show greater attention to stimuli such as beer bottles compared to non-problematic social drinkers (28).

These same patterns have also been found in behavioral addictions (20, 29), including among gamblers (30), video game players (31) and those addicted to the internet or social media (15, 32, 33). Specifically, in this case, Stanton Fraser et al. (34) investigated the use of social media-related images (logos of different platforms), confirming their role as salient attentional cues.

Neurophysiological evidence suggests that these users exhibit greater brain activation in response to this type of stimulus, manifested in earlier and heightened attention and a less efficient use of monitoring resources (35, 36). This suggests that social media content is more striking for this profile, influencing their attentional capacity and therefore their behavior.

A key question arising from this neurocognitive hyperreactivity observed in response to these specific stimuli is the possible existence of an underlying physiological sensitivity (at both the brain and bodily levels) that could be linked to individual predisposing factors.

Although this is a field that has yet to be thoroughly studied, there are already studies that discuss psychologically more vulnerable profiles (6). In these cases, social media use tends to be a response to maladaptive coping strategies, aimed at managing stress, loneliness or emotional distress (25, 37). In such cases, engagement with social media would be driven by distress, rather than from a purely behavioral addictive pattern which evolves from recreational use based on positive reinforcement towards a need sustained by negative reinforcement (38).

In recent years, research in behavioral medicine has increasingly focused on understanding how chronic and acute exposure to stress affects health outcomes (39). During exposure to stressors, the body’s physiological response systems are activated (40). These systems interact closely with the immune system and are therefore significantly involved in the onset and maintenance of pathological states (41).

Due to the still limited evidence, it remains difficult to establish the existence of a biological basis for social media use patterns; however, research is beginning to emerge that focuses on this possible predisposition, seeking links between physiological variables and social media use as compensatory strategies in response to negative emotional states or environmental stressors (42, 43).

Some of the most widely studied physiological markers are cardiovascular parameters and cortisol levels (44, 45) due to their ability to reflect the autonomic nervous system (ANS) and endocrine system, and their role in the physiological response to stress (46).

Specifically, among cardiovascular biomarkers, heart rate and heart rate variability are key indicators for assessing ANS activity (47). Heart rate variability (HRV) reflects the nervous system’s ability to adapt flexibly to environmental demands and has been associated with both emotional regulation and social stimuli (48). Furthermore, a review of studies on social interaction indicates that positive interactions (including online ones) have the potential to increase heart rate variability as an indicator of improved autonomic regulation (49).

Evidence on the physiological effects of social media use is mixed, with some studies reporting stress-attenuating effects in general users (50) and others finding that social media exposure can prolong activation of the stress axis following a psychosocial stressor (51), highlighting the importance of individual differences and specific patterns of use as key modulators of psychophysiological reactivity.

One framework that could account for this disparity is the stress-strain-outcome (SSO) model, mentioned earlier, which posits that certain individual factors, such as personality traits, act as predisposing factors for experiencing emotional strain in the face of environmental stressors, leading to negative behavioral consequences (10, 11). These factors would directly influence the way in which individuals perceive and process stimuli from social media (9), determining whether the user experiences well-being or, conversely, fatigue and psychological distress (stress). This may contribute to a predisposition to distress in the individual, potentially characteristic of this profile.

In this context, salivary hormones and enzymes emerge as particularly suitable tools for operationalizing and understanding physiological responses. However, empirical research has largely focused on the use of cortisol as the primary biomarker of stress (44, 45), which has limited consideration of other relevant biomarkers such as alpha-amylase or oxytocin.

Salivary alpha-amylase (sAA) has recently emerged as a valid and reliable marker of ANS activity in stress research and is therefore an important biomarker to consider in behavioral medicine (52). It has been established as a sensitive indicator of sympathetic system activation, reflecting rapid responses to psychological and social stimuli or challenges (52, 53). It has the distinct advantage of rising more rapidly than cortisol in the face of a threat or acute stressor (54).

It is therefore not surprising that current evidence suggests this marker is particularly reactive in individuals with psychological difficulties. Regarding social media use, the experiment by Shafi et al. (55) showed a significant increase in (sAA) levels in adolescents with depression, in contrast to the healthy control group, whose response was minimal and not statistically significant following 20 minutes of unrestricted exposure to social media.

Alongside the sympathetic activation reflected by alpha-amylase, oxytocin emerges as a key neuroendocrine marker for understanding the social dimension of physiological reactivity. Historically recognized for its role in attachment and interpersonal cooperation (56), oxytocin plays a crucial role in modulating emotional and social behavior and the formation of bonds, as it facilitates large-scale cooperation in human social networks by promoting prosocial behavior and the formation of interpersonal bonds (57).

Furthermore, this hormone plays a crucial role in stress regulation, facilitating a return to homeostasis following a psychosocial threat by increasing parasympathetic control and inhibiting activity in the amygdala (58–60), potentially promoting stress-attenuating processes. This includes a faster recovery from stress axis activation following a psychosocial challenge (61).

However, this effect is not inherently prosocial; rather, it appears to modulate sensitivity to the salience of social cues. This implies that its biological impact depends on both context and inter-individual factors (62) and may even promote defensive emotions or less adaptive stress responses if environmental stimuli are interpreted as unsafe or threatening. Furthermore, intranasal administration of oxytocin influences the functional connectivity of brain networks involved in the control of attention, emotion and reward, suggesting that oxytocin has a modulatory effect on interactions within and between neural networks (63).

Examples include the studies by Cardoso et al. (64) and Quirin et al. (65), in which intranasal administration of oxytocin reduced the subjective stress response and cortisol levels only in individuals with poor coping skills or emotional regulation, respectively, but not in those with adequate skills.

In the context of digital technology use, this pattern may carry particular relevance for identifying vulnerable profiles, as this potential biological limitation could leave certain users unprotected against stress, positioning oxytocin as a key marker for identifying profiles with greater physical and emotional vulnerability to digital stimuli and as a potential determinant of adaptive flexibility, influencing whether such interactions mitigate the stress response or, conversely, contribute to the autonomic rigidity observed in more vulnerable individuals.

In this regard and given that previous studies have relied predominantly on self-reports (66), which constitutes a significant methodological limitation, it is necessary to incorporate objective biomarkers that allow these cognitive processes to be linked to physiological responses more accurately (50). For this reason, the present study aims to overcome the limitations of behavioral methods through a multidimensional, real-time assessment, using an adapted Go/Nogo paradigm with social media stimuli and simultaneously measuring heart rate variability (SDNN), salivary alpha-amylase and oxytocin levels, with the central objective of understanding the interaction between cognitive control, stress regulation and the psychophysiological response to digital stimuli, differentiating user profiles.

Based on this theoretical framework, the present study tested the following hypotheses. First, we hypothesized that the functional profile would show superior inhibitory control performance and more efficient autonomic recovery (greater post-task SDNN increase) compared to the compulsive use and distress-associated profiles. Second, we hypothesized that the distress-associated profile would exhibit the greatest inhibitory control deficits, particularly in response to social media-specific stimuli, alongside reduced autonomic flexibility evidenced by smaller SDNN changes across phases. Third, we hypothesized that salivary alpha-amylase and oxytocin would show differential reactivity patterns across profiles, with the compulsive use profile expected to display heightened sympathetic reactivity during task exposure followed by efficient recovery, while the distress-associated profile was expected to show blunted or atypical biomarker responses consistent with dysregulated stress processing.

## Materials and methods

**Figure 1:**
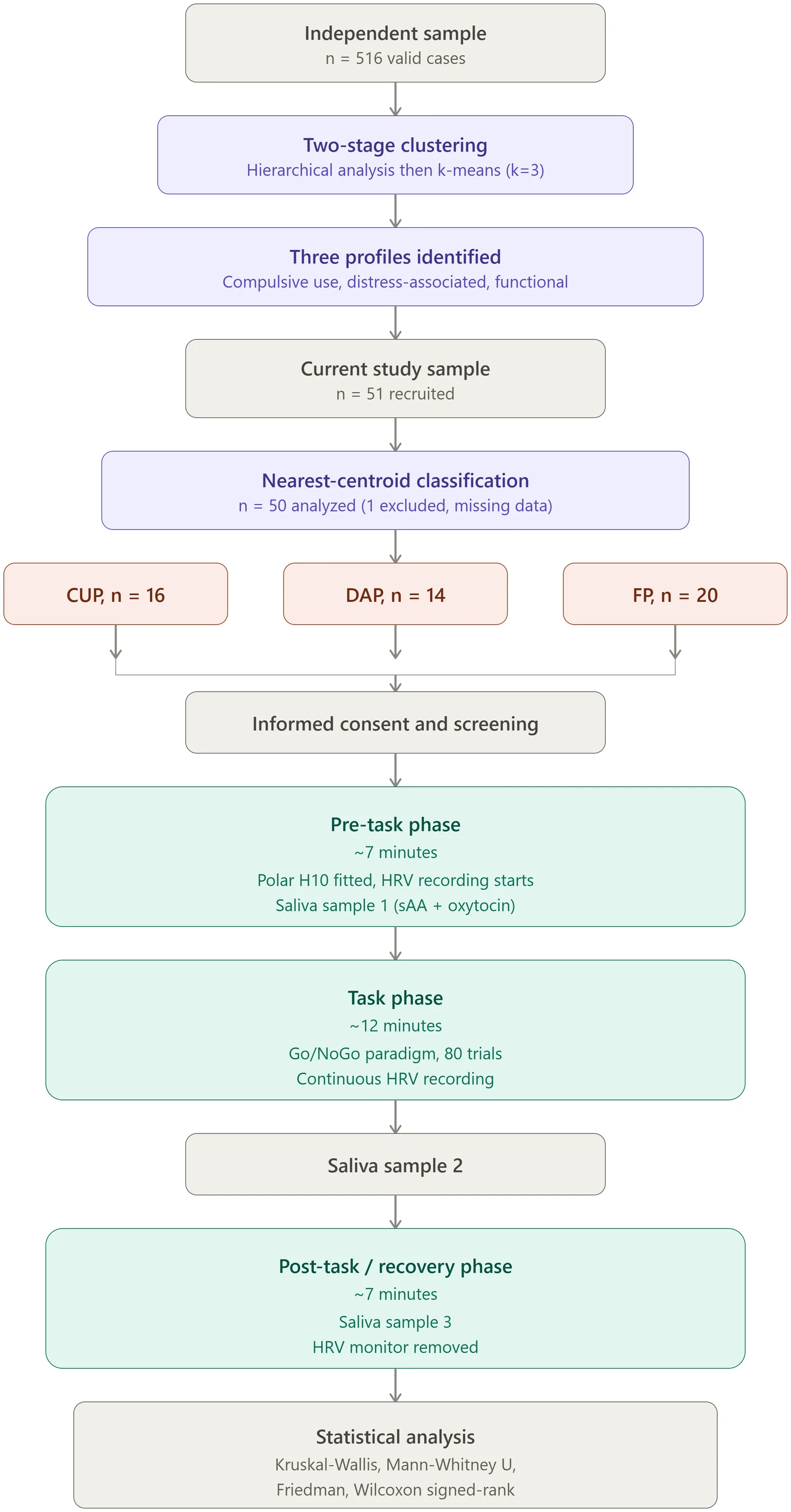
Study flowchart depicting participant recruitment from the independent clustering sample, cluster assignment procedure, and the three-phase experimental protocol with corresponding biomarker collection points.

The overall study design, including participant recruitment, cluster assignment, and the three-phase experimental protocol, is summarized in **Figure 1**.

### Participants

The study included a total of 50 participants with a mean age of 19.22 years and a standard deviation of 1.73. The age range was between 18 and 25 years. 59.2% were women (n=29). Furthermore, 6.1% were studying at sixth-form or intermediate level, 89.8% were studying for a bachelor’s degree, and the remaining 4.1% were studying for a master’s degree. The majority of participants were students at Francisco de Vitoria University, Madrid, Spain.

### Cluster assignment procedure

Participant profiles were derived using a two-stage clustering approach to ensure robustness. In the first exploratory stage, a hierarchical cluster analysis was conducted using SPSS v.25, and visual inspection of the dendrogram suggested a three-cluster solution as optimal. In the second confirmatory stage, a k-means algorithm (k = 3) was applied to definitively assign each case based on proximity to cluster centroids, using five clustering variables: total score on the problematic smartphone and social media use scales (PSNUSS), daily hours of social media use, total score on the depression anxiety stress scales (DASS), total score on the obsessive-compulsive-related disorder use scale (OCDUS-TIC), and the emotional regulation subscale of the Wong-Law Emotional Intelligence Scale (WLEIS-RegEm). The analysis was conducted on an independent sample (n = 516 valid cases) and convergence was achieved after 6 iterations (maximum absolute coordinate change = .000; minimum distance between initial centers = 69.788).

The discriminant validity of the clustering variables was evaluated through ANOVA-based variance explained (R²): PSNUSS showed the highest discriminant power (R² = .66), followed by OCDUS (R² = .49), DASS (R² = .31), WLEIS-RegEm (R² = .10), and daily hours of use (R² = .05). Separation between final cluster centers ranged from 24.47 to 31.50, indicating adequate between-profile differentiation. Cluster solution stability was confirmed by inspecting the iteration history, with convergence achieved after six iterations and no further centroid displacement.

External normative criteria were used to interpret profile severity: DASS scores were contextualized using established severity ranges (0–20 very low; 21–40 mild; 41–60 moderate; 61–80 high; >80 very high), and PSNUSS scores were referenced against the original validation mean of 3.12.

The final cluster centers were as follows: Cluster 1 (CUP, compulsive use profile; n = 131): PSNUSS = 84.13, hours per day = 3.67, DASS = 22.37, OCDUS = 58.56, WLEIS-RegEm = 18.14; Cluster 2 (DAP, distress-associated profile; n = 164): PSNUSS = 66.13, hours per day = 3.43, DASS = 27.56, OCDUS = 47.13, WLEIS-RegEm = 17.51; Cluster 3 (FP, functional profile; n = 221): PSNUSS = 51.33, hours per day = 2.66, DASS = 11.01, OCDUS = 36.23, WLEIS-RegEm = 21.01.

The participants in this study (n = 50) were assigned to profiles using the nearest-centroid clustering method, whereby each participant was assigned to the group whose final centroid was closest in Euclidean distance, based on their scores on the five clustering variables. This procedure is detailed in the (**Supplementary Table 1**).

The following profile assignments were made: 16 participants in the Compulsive Use Profile (CUP), 14 in the Distress-Associated Profile (DAP), and 20 in the Functional Profile (FP).

The study was approved by the Ethics Committee of Francisco de Vitoria University in accordance with the Ethical Principles for Medical Research Involving Human Subjects adopted in the Declaration of Helsinki by the World Medical Association (64th WMA General Assembly, Fortaleza, Brazil, October 2013), Recommendation No. R (97) 5 of the Committee of Ministers to Member States on the Protection of Medical Data (1997) and Organic Law 15/1999 on Data Protection (LOPD).

All participants were informed of the purpose and characteristics of the study prior to inclusion and subsequently gave their written informed consent.

### Procedure

Participants signed an informed consent form and were assigned an anonymized alphanumeric code to ensure confidentiality throughout data processing and analysis.

Participants who confirmed their attendance received a reminder 24 hours prior to the session, specifying the approximate duration (40 minutes) and pre-experimental instructions (fasting from liquids and solids and abstaining from strenuous physical exercise and smoking for at least one hour prior to the experiment). Upon arrival at the laboratory, compliance with these criteria was verified. All sessions were conducted between 12:00 and 18:00 to minimize circadian variability in cortisol-related biomarkers.

The experiment consisted of three key stages: first, the baseline or pre-task phase (pre), lasting approximately 7 minutes; next, the task phase, lasting an average of 12 minutes, during which the subjects performed a task under the Go/NoGo paradigm; and finally, the recovery or post-task phase (post), lasting approximately 7 minutes.

Upon arrival at the room where the experiment was conducted, participants were asked to sign the informed consent form, and the Polar H10 heart rate monitor was fitted, thus commencing continuous recording during the baseline phase (pre-phase), the task phase, and the recovery phase (post-phase).

Following a baseline period in a seated position, the first saliva sample (pre-phase) was collected using Salivette Cortisol devices (Sarstedt, S.A.U., La Roca del Vallès, Barcelona, Spain) with synthetic fiber swabs, which remained in the oral cavity for 2 minutes without chewing movements, for subsequent analysis of alpha-amylase and oxytocin. This concluded the first phase.

In the second phase, participants performed the Go/NoGo task (average duration ≈ 12 minutes). First, participants completed two practice trials to familiarize themselves with the experiment. This was followed by four rounds of 20 trials each, making a total of 80 trials. The task followed the same procedure and software as the original version (43), with the exception of its translation from German into Spanish and the replacement of Facebook stimuli with Instagram, given its greater ecological validity for the target population (1). All experimental images/sounds are included in the **Supplementary Material**.

Immediately after completing the test, the second saliva sample was collected using the same procedure.

Finally, during the recovery or post-task phase, the subject was asked to relax and, 10 minutes after the task had ended, the third saliva sample was collected using the same procedure and the heart rate monitor was removed, which had been used to measure heart rate throughout the experiment.

The experiment was conducted in a room with uniform lighting and controlled environmental conditions. All participants followed a standardized protocol, keeping the same spatial arrangement, distance from the screen, use of headphones and light levels, to minimize environmental variability.

### Measures/stimuli

#### Go/NoGo task

A modified auditory Go/NoGo paradigm using social media-related cues was employed to assess specific inhibitory control in response to digitally relevant stimuli. The task was adapted from the auditory paradigm developed by Wegmann et al. (67), further incorporating the conceptual principles of the Go/NoGo taxonomy proposed by (68). The methodological adaptation subsequently published in Scientific Reports (43) was also followed.

**Figure 2:**
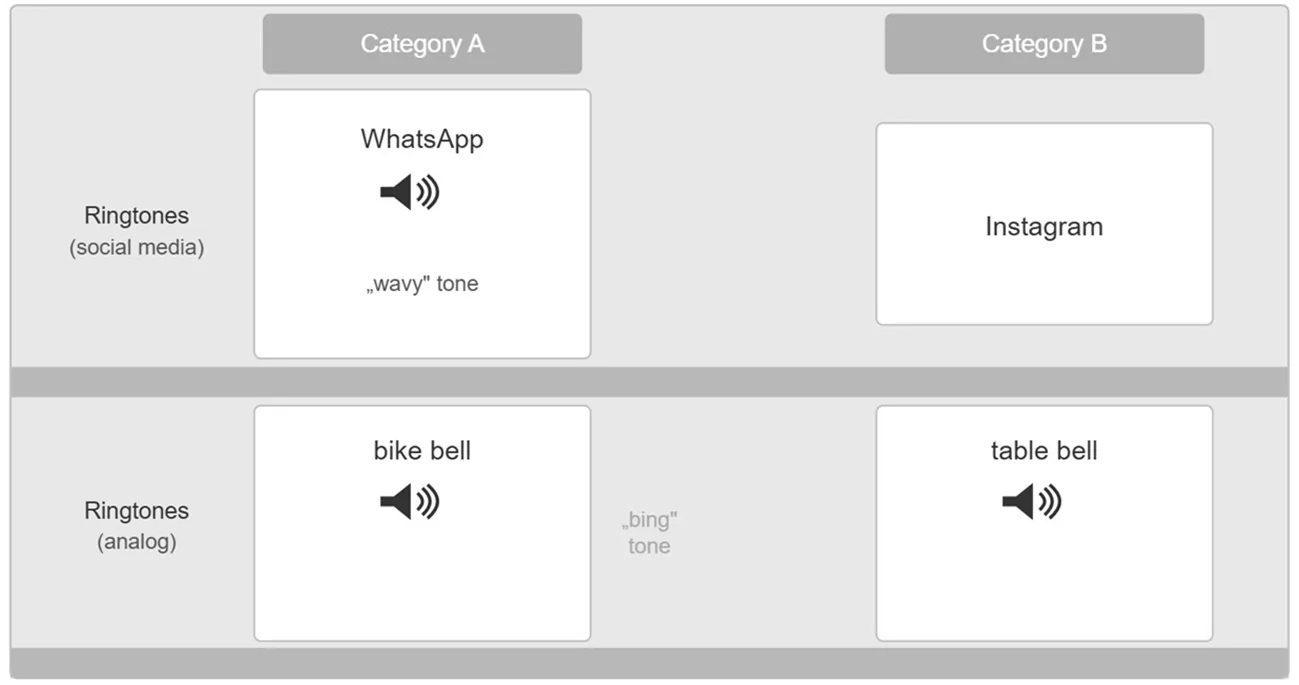
Categorization matrix of auditory stimuli used in the experiment. Visualization of the cues used in the auditory Go/NoGo task, including social-network-related ringtones. Each category contains two stimuli with different attributes (social media vs. analog; “wavy” vs. “bing” tone). The presentation of the stimuli was pseudorandomized (see also 43, 67).

Four auditory ringtones of comparable duration were used (**Figure 2**). Two corresponded to digital stimuli related to social media (standard notification tones from Instagram and WhatsApp). The switch from Facebook to Instagram in the task was designed to increase the ecological validity of the paradigm, given that this social media is a relevant and highly salient digital stimulus in the participants’ daily lives (1). The other two ringtones corresponded to neutral analogue stimuli (a doorbell and a bicycle bell). The analogue stimuli were carefully selected to maintain systematic acoustic similarities with the digital tones, following the methodological criteria described in previous studies (67, 68).Both the training phase and the actual task phase are described in the original study (43).

Participants had to respond as quickly as possible to Go stimuli and refrain from responding to NoGo stimuli. Measures of hits, false alarms, misses, and reaction times were recorded, distinguishing between social media-related stimuli and neutral stimuli.

For the analysis of performance in the Go/NoGo task, the data were categorized according to the Signal Detection Theory (SDT) framework, which allows for a more precise interpretation of the efficiency of inhibitory control and the participants’ response bias.

Under the Go/NoGo adaptation of the SDT framework: hits refer to correct responses to Go stimuli (responding when required); misses refer to failures to respond to Go stimuli (omissions); false alarms refer to responses made to NoGo stimuli (commission errors reflecting failed inhibition); and correct rejections refer to successful inhibition of responses to NoGo stimuli.

#### Heart rate

Heart rate variability (HRV) was recorded using a Polar H10 heart rate monitor (Soluciones Deportivas, S.L., Madrid, Spain), a device validated for use in psychophysiological research (sampling rate: 1000 Hz; RR interval recording precision: 1 ms). The recording was continuous throughout the experiment (pre-task, task and post-task phases).

Later signal processing and analysis were carried out using Kubios HRV software (Kubios Oy, Kuopio, Finland), widely used for heart rate variability (HRV) analysis. The standard artefact correction and automatic filtering procedures recommended by the software were applied.

The following indices were analyzed in the time domain: SDNN (Standard Deviation of NN intervals): standard deviation of all NN intervals, which reflects overall heart rate variability and was selected as the primary HRV outcome given its sensitivity to overall autonomic modulation across short-term recordings and its widespread use in comparable experimental paradigms (69). RMSSD (Root Mean Square of the Successive Differences): the root mean square of the successive differences between adjacent normal RR intervals (ms), considered a sensitive marker of parasympathetic activity, was analyzed as a secondary index. Both indices are reported in the Results section.

These metrics enabled the assessment of autonomic regulation and the psychophysiological changes associated with the performance of the experimental task.

#### Oxytocin

Oxytocin levels were determined using a direct competitive assay based on AlphaLISA technology (PerkinElmer, MA, USA), following previously validated protocols (70).

The acceptor microspheres were coated with an anti-oxytocin monoclonal antibody, whilst streptavidin-conjugated G-protein-bound microspheres were used as donor microspheres. To optimize the assay, different concentrations of biotinylated oxytocin, acceptor microspheres and donor microspheres were evaluated, and the conditions offering the highest sensitivity and analytical stability were selected. The standard curve was prepared using oxytocin conjugated with bovine serum albumin (BSA).

Salivary oxytocin was measured using a monoclonal antibody-based immunoassay, analytically validated for human saliva by López-Arjona et al. (71) at the same laboratory (Interlab-UMU, University of Murcia) where the present samples were processed. This assay was selected over polyclonal alternatives based on its more clearly defined epitope binding region, which confers greater specificity. It should nonetheless be acknowledged that salivary oxytocin measurement remains methodologically debated in psychobiological research, given ongoing discussion regarding the relationship between peripheral (salivary) and central oxytocin activity, as well as variability in reported concentrations depending on the assay and antibody type used (71, 72).

#### Alpha-amylase

Salivary alpha-amylase activity was quantified using a kinetic spectrophotometric assay following the method recommended by the International Federation of Clinical Chemistry and Laboratory Medicine, by previously described protocols (73).

The procedure used 4,6-ethylidene(G7)-p-nitrophenol(G1)-alpha-D-maltoheptoside (ethylidene-G7PNP) as the enzymatic substrate. The intermediate product generated following hydrolysis reacted with alpha-glucosidase to produce p-nitrophenol as the final product. The rate of p-nitrophenol formation, proportional to enzymatic activity, was determined by spectrophotometric measurement at 405 nm.

The analysis was performed on an Olympus AU2700 automatic analyzer (Olympus America Inc., Center Valley, PA, USA).

### Statistical analysis

IBM SPSS Statistics version 25 was used to process the data, with a significance level set at α = .05.

Firstly, the normality of the variables was assessed using the Shapiro-Wilk test, given the small size of the groups. As normality was not met in the majority of variables and groups, non-parametric tests were employed throughout.

It should be noted that physiological analyses were conducted on subsamples of the total analyzed sample (n = 50) due to missing data arising from technical issues during recording or insufficient saliva volume. Specifically, HRV analyses (SDNN and RMSSD) were conducted on n = 40 participants due to signal quality issues or equipment fitting problems during recording; oxytocin analyses on n = 45–48 participants; and alpha-amylase analyses on n = 46–49 participants, with missing data in the latter two variables due to insufficient saliva samples or processing issues. Complete case analysis was used for each variable independently.

To analyze changes over time within each cluster, the Friedman test for paired samples was used. When the overall comparison proved significant, *post hoc* tests were conducted using the Wilcoxon test with Bonferroni correction to adjust the significance level. To directly examine whether psychophysiological response patterns across the three experimental phases differed between profiles, Scheirer-Ray-Hare non-parametric two-way tests were conducted for each physiological variable (SDNN, MeanHR, salivary alpha-amylase, and oxytocin). This test was selected given the violations of normality observed across multiple groups and variables and provides a direct non-parametric assessment of the Profile × Phase interaction effect.

Between-group differences in Go/No-Go performance variables were examined using Kruskal-Wallis tests, with effect sizes calculated as η² = (H − k + 1)/(n − k). Significant omnibus results were followed by Mann-Whitney U *post-hoc* comparisons with Bonferroni correction (α’ = .017 for three pairwise comparisons). The sensitivity index d’ was calculated using the Hautus correction: d’ = Z (hits + 0.5)/(hits + misses + 1) − Z(false alarms + 0.5)/(false alarms + correct rejections + 1). Within-subject SNS-versus-neutral comparisons were conducted using Wilcoxon signed-rank tests within each profile separately. Between-group differences in RMSSD were examined using a Kruskal-Wallis test across the three profiles.

## Results

### Go/No-Go test

Go/No-Go task performance was examined across two domains: general inhibitory control variables and variables specific to social media (SNS) stimuli. **Tables 1** and **2** present the descriptive analyses of all the variables measured.

**Table 1:** Differences between clusters in general Go/No-go task measures.

Measure	CUP (n=16)	DAP (n=14)	FP (n=20)	Total (n=50)	H (2)	p	η²
Hits	37.06 (4.88)	36.71(4.43)	38.80 (1.47)	37.66 (3.77)	1.33	.514	.000
Correct Rejection	36.38 (6.63)	35.29 (3.62)	37.95 (1.73)	36.70(4.40)	6.16	.046*	.089
False Alarm	2.94 (4.88)	3.29 (4.43)	3.20 (2.52)	2.36 (3.77)	5.14	.076	.067
Misses	3.56 (6.39)	4.71 (3.63)	2.00 (1.71)	3.26 (4.29)	6.46	.039*	.095

H, Kruskal–Wallis statistic; df, 2 in all cases; *, Significant values (p <.05); η², Kruskal-Wallis effect size; CUP, Compulsive Use Profile; DAP, Distress-Associated Profile; FP, Functional Profile.

**Table 2:** Differences between clusters in specific variables for social media stimuli (SNS).

Measure	CUP (n = 16)	DAP (n = 14)	FP (n = 20)	TOTAL (n=50)	H (2)	p	η²
Misses_SNS	2.25 (3.59)	3.00 (2.15)	1.20 (1.15)	2.04 (2.50)	6.36	.042*	.093
FalseAlarm_SNS	1.63 (2.78)	1.71 (2.30)	0.65 (1.04)	1.26 (2.11)	2.19	.335	
Correct Rejections SNS	17.69 (3.79)	17.00 (2.15)	18.80 (1.15)	17.94 (2.59)	6.36	.042*	.093
Reaction Speed SNS (ms)	1048.81 (136.94)	996.71 (120.01)	1047.90 (176.98)	1033.86 (149.18)	.72	.699	

H, Kruskal–Wallis statistic; df, 2 in all cases; *, Significant values (p <.05); η², Kruskal-Wallis effect size.

Descriptive statistics for general performance variables indicated that the FP achieved the highest number of correct responses and the lowest error rates across all measures, followed by the CUP and the DAP.

Analysis using the Kruskal-Wallis test revealed no significant differences between the groups in the number of correct responses (H (2) = 1.33, p = .514), indicating comparable overall performance across profiles in the general execution of the task. However, significant between-group differences emerged for omissions (H (2) = 6.47, p = .039) and correct rejections (H(2) = 6.17, p = .046).

Effect sizes for the significant Kruskal-Wallis tests, calculated as η², indicated small-to-moderate effects for Misses (η² = .095) and Correct Rejections (η² = .089) in the general task, and for Misses SNS (η² = .093) and Correct Rejections SNS (η² = .093) in the social-media-specific condition (see **Tables 1**, **2**).

*Post-hoc* tests using the Mann-Whitney U test with Bonferroni correction (α = .017) revealed that differences in omissions were found exclusively between the DAP and FP groups (U = 72.50, Z = -2.40, p = .017), with the DAP group having the highest number of omissions (M = 4.71, SD = 3.63) compared to the FP group (M = 2.00, SD = 1.72). As for correct rejections, the comparison between DAP and FP showed a trend close to the significance threshold after correction (U = 75.00, Z = -2.30, p = .021), whilst comparisons between CUP and DAP (U = 63.50, Z = −2.04, p = .043) and CUP and FP (U = 158.50, Z = −0.05, p = .961) did not reach the corrected significance threshold.

Turning to the social media-specific variables, descriptive statistics again favored the FP, which exhibited lower error rates across omissions and false alarms and higher correct rejection rates (**Table 2**).

In this case, the analyses again revealed significant differences in omissions (H_2_ = 6.36, p = .042) and in correct SNS rejections (H_2_ = 6.36, p = .042), with *post-hoc* analyses again revealing that both effects were driven by the DAP vs. FP comparison. The DAP group showed significantly more SNS omission errors (U = 69.00, Z = -2.55, p = .011) and fewer correct SNS rejections (U = 69.00, Z = -2.55, p = .011) than the FP group, with no significant differences found in the remaining pairwise comparisons.

A complementary within-subject analysis was conducted to examine whether inhibitory control deficits were specific to social media stimuli or reflected a general inhibitory pattern. Wilcoxon signed-rank tests comparing SNS-specific and neutral stimulus conditions within each profile revealed that only the DAP showed statistically significant differences between conditions: this profile committed significantly more omissions (Z = -2.196, p = .028) and fewer correct rejections (Z = -2.196, p = .028) in response to SNS stimuli compared to neutral stimuli. No significant SNS-versus-neutral differences were found in the CUP (omissions: Z = -1.652, p = .098; correct rejections: Z = -1.760, p = .078) or the FP (omissions: Z = -1.642, p = .101; correct rejections: Z = -1.393, p = .163). False alarm rates did not differ significantly between SNS and neutral conditions in any profile (CUP: p = .491; DAP: p = .739; FP: p = .681).

In addition, participants’ discrimination ability was assessed by calculating the sensitivity index (d’), both in general and in response to specific social media stimuli (**Table 3**).

**Table 3:** Descriptive statistics and Kruskal-Wallis test results for general d’ and SNS d’ by group.

Measure	CUP (*n* = 16)	DAP (*n* = 14)	FP (*n* = 20)	H (2)	p	η²
General d’	3.33 (1.73)	3.18 (1.34)	3.73 (1.24)	1.33	.514	.000
SNS d’	2.99 (1.76)	2.91 (1.33)	3.44 (1.26)	2.19	.335	.004

Values are expressed as M (SD). SNS d’, discriminative sensitivity specific to social media stimuli. H (2) corresponds to the Kruskal-Wallis test with 2 degrees of freedom. η², Kruskal-Wallis effect size. CUP, Compulsive Use Profile; DAP, Distress-Associated Profile; FP, Functional Profile.

The descriptive results show that the functional profile exhibited the highest discriminative sensitivity in both the general task and the social media-specific stimuli, followed by the compulsive use profile. In contrast, the profile associated with distress showed the lowest sensitivity values in both categories.

Despite these differences observed in the means, statistical analysis using the Kruskal-Wallis test did not reach the conventional level of significance, neither for the general d’ (H(2) = 1.33, p = .514, η² = .000) nor for the social media d’ (H(2) = 2.19, p = .335, η² = .004), indicating negligible effect sizes.

### Heart rate

Heart rate variability was assessed using the MeanHR variable (mean heart rate), measured in beats per minute (bpm), which provides an overview of overall cardiac activity. The SDNN index (standard deviation of NN intervals), expressed in milliseconds (ms), was used as an overall indicator of autonomic regulation.

**Figure 3:**
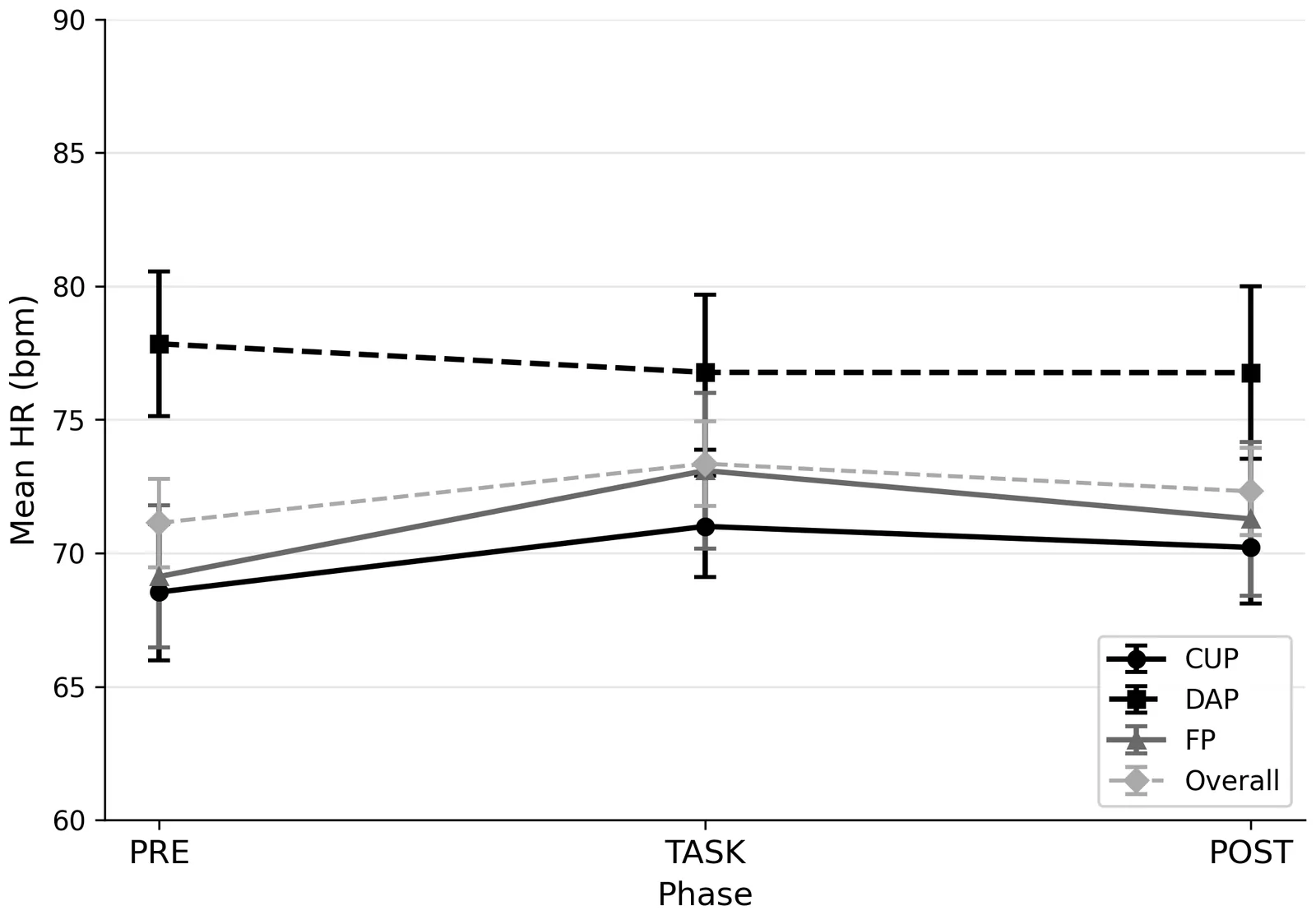
Mean heart rate (MEAN_HR, bpm) per cluster and overall mean across the three experimental phases (Pre, Task, Post). Error bars represent standard error of the mean (SE). CUP, Compulsive Use Profile; DAP, Distress-Associated Profile; FP, Functional Profile.

Regarding MeanHR, As illustrated in **Figure 3**, descriptive statistics indicated an increase in heart rate levels during the task, which decreased in the post-task phase, generally returning to baseline levels in both the functional and compulsive use profiles. However, it is worth noting that the DAP group showed virtually no differences between time periods and always exhibited higher heart rate levels than the other groups.

Despite these findings, these differences did not reach statistical significance in the comparative analyses. The Friedman test did not reveal any significant changes over time in any of the profiles; FP, χ² (2) = 4.11, p = .128; DAP, χ² (2) = 0.80, p = .670; and CUP, χ² (2) = 4.50, p = .105 (**Table 4**).

**Table 4:** Intra-group statistics on physiological measures across experimental phases by cluster (Friedman test and *post hoc* comparisons with Bonferroni correction).

Measure	Cluster (N)	Pre	Task	Post	χ² (2)	p	Significant *post hoc* (Bonferroni)
MeanHR	FP (18)	69,11(11,27)	71,51(10,96)	69,77(11,08)	4.11	.128	—
	DAP (10)	77.84 (8.59)	78,13 (8,98)	78,36 (9,80)	0.80	.670	—
	CUP (12)	68,54 (8.82)	70.91 (7.61)	69,22 (7.98)	4.50	.105	—
SDNN	FP (18)	69.96 (29.87)	64,30 (26,67)	71,52 (31,56)	7.11	.029*	Task < Post (Z = −3.02, p = .003)
	DAP (10)	58.75 (36,28)	57.34 (22,53)	58,62 (26,12)	0.80	.670	—
	CUP (12)	61.15 (25.78)	56,57 (17,14)	64,80 (25,88)	7.16	.028*	Task < Post (Z = −2.13, p = .033)
Oxytocin (pg/mL)	FP (18)	1.57 (1.23)	1.62 (1.32)	1.93 (1.50)	0.187	.911	—
	DAP (12)	1.58 (1.06)	2.01 (1.31)	2.65 (3.74)	2.16	.338	—
	CUP (15)	1.62 (1.50)	2.00 (1.91)	1.67 (2.01)	1.73	.420	—
Alpha-amylase (kUI/L)	FP (19)	344.43 (278.31)	248.69 (154.17)	216.18 (171.77)	6.00	.050*	Pre < Post (Z = −3.28, p = .001)
	DAP (12)	236.74 (145.42)	200.38 (96.77)	213.05 (124.06)	0.50	.779	—
	CUP (15)	219.25 (92.28)	242.94 (129.38)	184.81 (102.38)	2,13	.344	—

Values are expressed as M (SD). χ² = Friedman test statistic (df = 2). Post hoc comparisons were conducted using the Wilcoxon signed-rank test for related samples. *p <.05.

**Figure 4:**
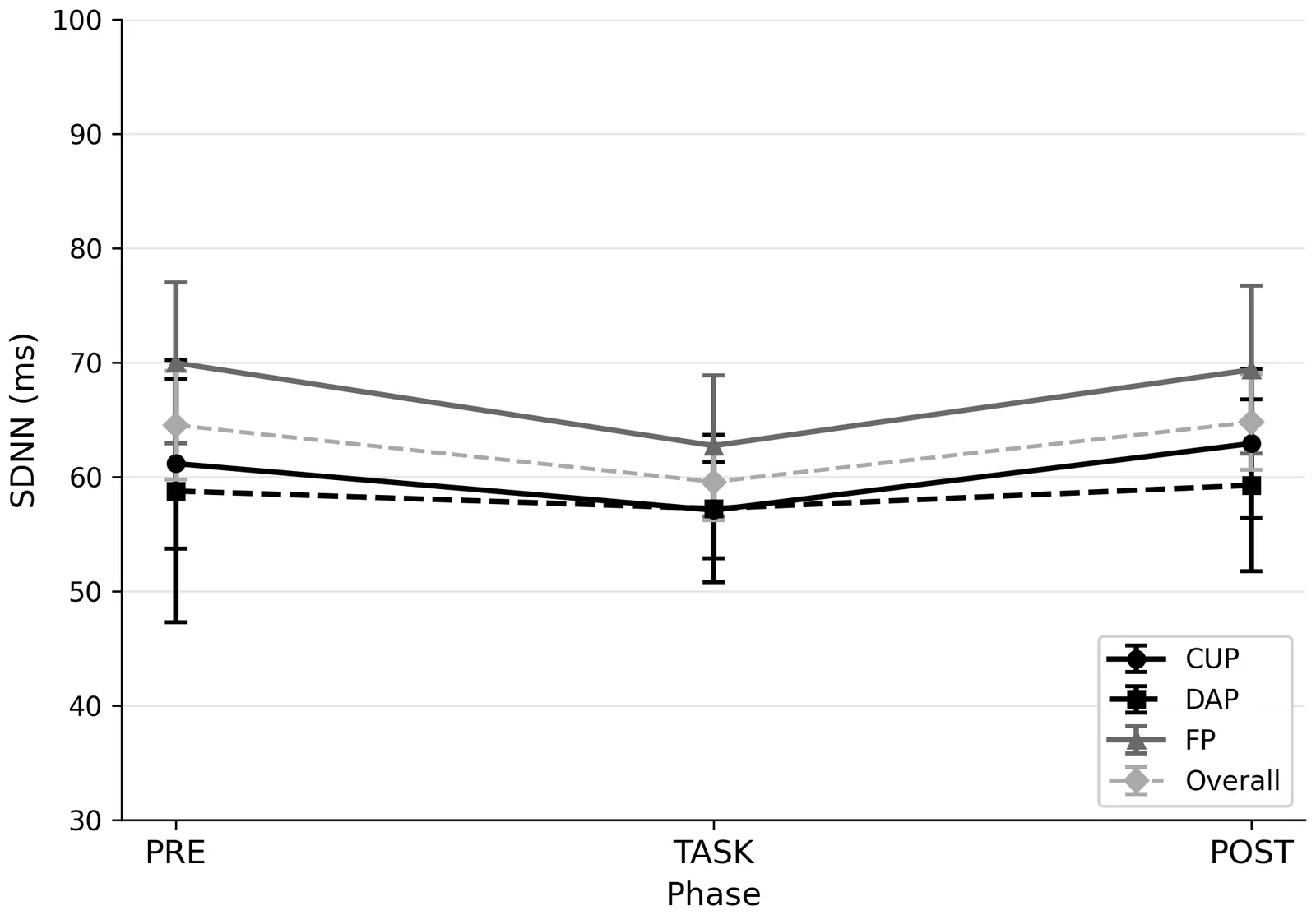
SDNN (ms) per cluster and overall mean across the three experimental phases. Error bars represent standard error of the mean (SE). CUP, Compulsive Use Profile; DAP, Distress-Associated Profile; FP, Functional Profile.

Regarding the SDNN measure, descriptive statistics showed a decrease in SDNN during the experimental task compared to the baseline phase in all profiles, which subsequently increased during the post-task phase (**Figure 4**).

Overall, the compulsive use profile showed the greatest autonomic reactivity (the greatest decrease from the baseline phase), whilst the distress-associated profile exhibited lower overall cardiac variability and reduced reactivity, again showing less variation over time. The functional profile showed a pattern of greater stability and autonomic recovery following the task.

These differences reached statistical significance in the comparative statistics, as shown in **Table 4**.

The Friedman test indicated significant differences between the three measurement points, χ² (2) = 7.11, p = .029 in the FP, with a significant increase in SDNN observed from the task to the post-task period in the Wilcoxon *post-hoc* test (Z = −3.02, p = .003). No significant differences were observed between the remaining phases: baseline-task (Z = −1.72, p = .085) and baseline-recovery (Z = −1.067, p = .286). This pattern suggests effective autonomic recovery following task-induced activation.

Meanwhile, in the compulsive use profile (n = 12), the Friedman test also revealed significant differences between the three time points, χ² (2) = 7.16, p = .028. *Post-hoc* comparisons using Wilcoxon tests indicated an increase in SDNN from the task period to the post-task period (Z = −2.13, p = .033); however, this result does not survive Bonferroni correction for three pairwise comparisons (α’ = .017) and should therefore be interpreted with caution. Whilst no significant differences were observed between the baseline and task periods (Z = −0.628, p = 0.53) or between baseline and post-task (Z = −1.64, p = 0.99).

In the DAP, the Friedman test showed no significant differences between time points, χ² (2) = 0.80, p = .670, indicating stability in heart rate variability throughout the experimental protocol.

RMSSD values showed a descriptively similar pattern across the three phases in all profiles (CUP: Pre = 98.81, Task = 59.18, Post = 60.57; DAP: Pre = 62.98, Task = 61.47, Post = 59.95; FP: Pre = 71.68, Task = 64.39, Post = 66.22). However, Friedman tests revealed no statistically significant differences between measurement phases in any profile (CUP: χ²(2) = .167, p = .920, n = 12; DAP: χ²(2) = .800, p = .670, n = 10; FP: χ²(2) = 1.333, p = .513, n = 18). No significant between-group differences in RMSSD were identified (Kruskal-Wallis: H (2) = 2.077, p = .354, η² = .002).

To directly assess whether the pattern of physiological change across phases differed between profiles, Scheirer-Ray-Hare tests were conducted for each variable. Results indicated no significant Profile × Phase interaction for SDNN (H (4) = 0.553, p = .968), alpha-amylase (H (4) = 1.390, p = .846), or oxytocin (H (4) = 1.749, p = .782). For MeanHR, no significant Profile × Phase interaction was found (H (4) = 0.200, p = .995); however, a significant main effect of Profile was detected (H (2) = 17.584, p <.001), indicating that the three profiles differed significantly in their overall mean heart rate levels across the experiment, with the DAP exhibiting consistently higher values than the CUP and FP. These findings indicate that whilst within-profile phase changes were observed for SDNN in the CUP and FP, the magnitude of these changes did not differ significantly between profiles.

### Oxytocin

**Figure 5:**
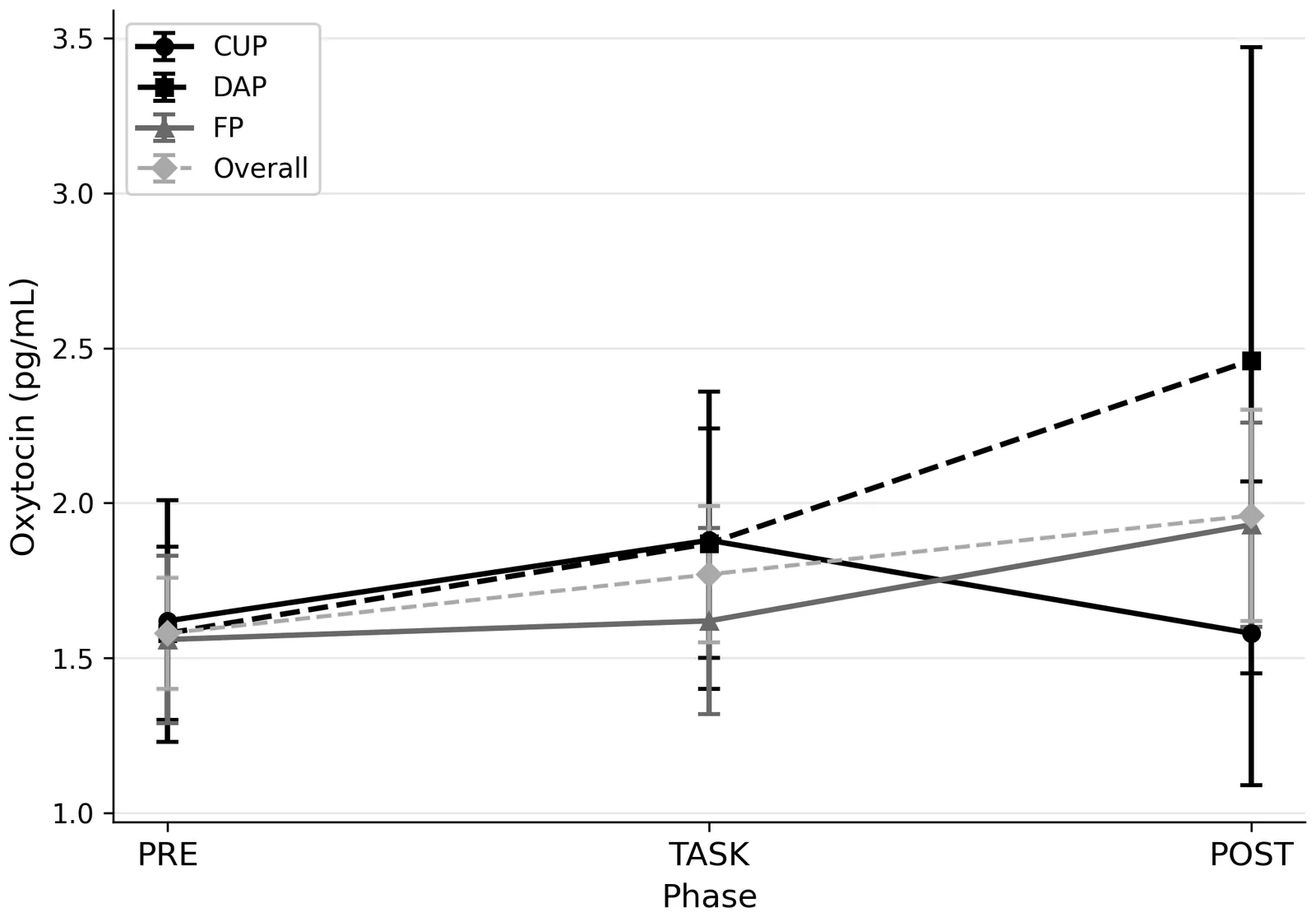
Oxytocin levels (pg/mL) per cluster and overall mean across the three experimental phases. Error bars represent standard error of the mean (SE). CUP, Compulsive Use Profile; DAP, Distress-Associated Profile; FP, Functional Profile.

The mean oxytocin levels for the overall sample, shown in **Figure 5**, exhibited a slight upward trend throughout the experimental protocol.

By cluster, high inter-individual variability was observed at all measurement time points. Standard deviations were high across all profiles, with markedly asymmetric distributions and the presence of outliers, indicating considerable heterogeneity in hormone levels.

An increase in oxytocin was observed across all profiles between the pre-task and task phases, with more pronounced increases in the CUP and DAP. However, diversity was again found between the task and post-task phases, whilst FP and DAP showed a continued rise in levels, particularly pronounced in the profile associated with distress. The compulsive profile was the only one to show a decrease in this measure following the task.

Despite these descriptive results, none of the three profiles showed significant differences following the Friedman test. The results are presented in **Table 4**.

### Alpha-amylase

In the total sample, alpha-amylase levels showed a progressive increase throughout the protocol (Pre: M = 1.59; Task: M = 1.77; Post: M = 1.95). An overall increase was observed from the baseline phase to the post-task phase in the sample as a whole.

**Figure 6:**
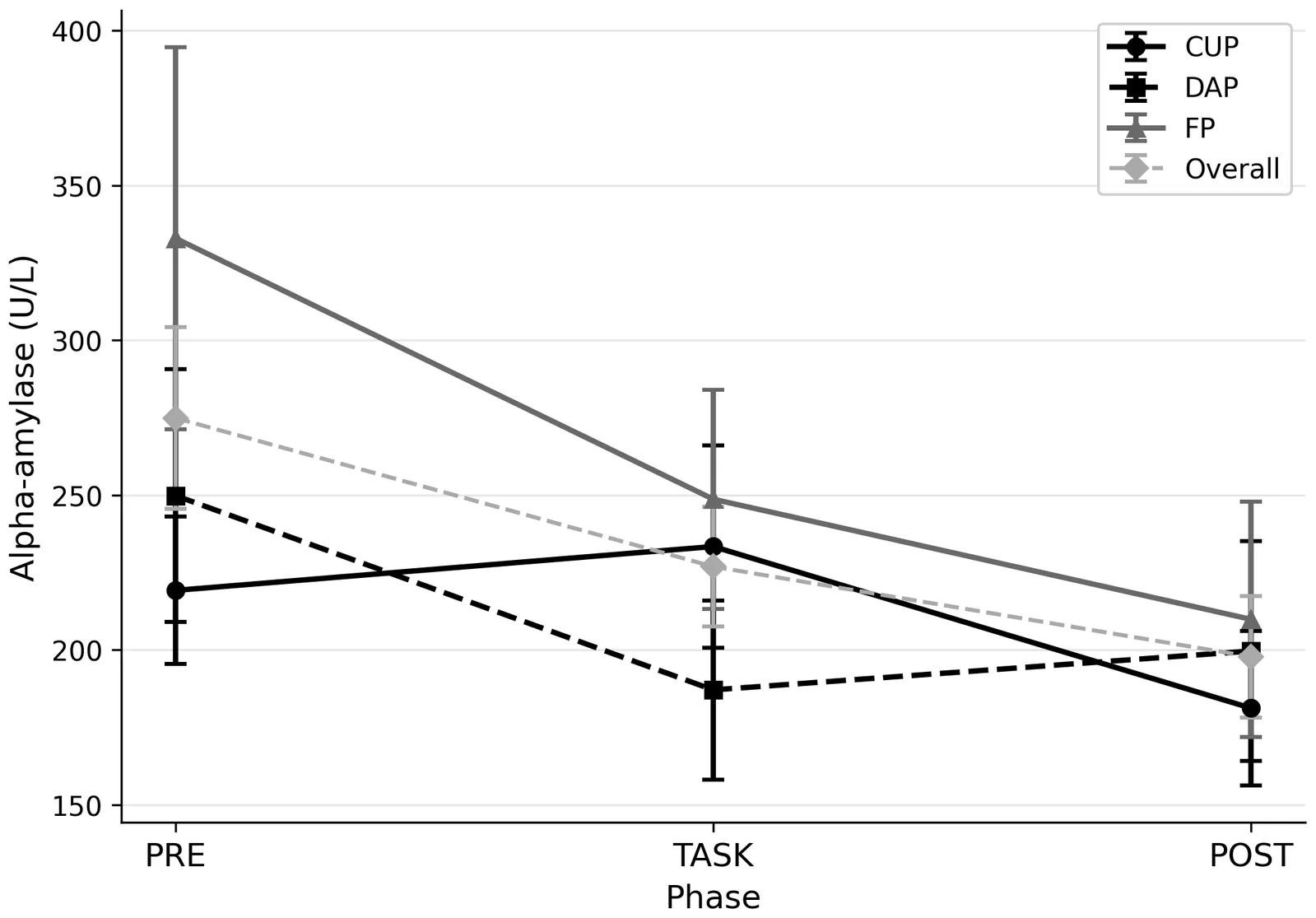
Alpha-amylase levels (kUI/L) per cluster and overall mean across the three experimental phases. Error bars represent standard error of the mean (SE). CUP, Compulsive Use Profile; DAP, Distress-Associated Profile; FP, Functional Profile.

In the cluster-based descriptives (**Figure 6**), high inter-individual variability was observed at all measurement points.

In the baseline phase, the FP profile had the highest mean, followed by the DAP and the CUP.

The latter profile is characterized as the only one to show an increase in levels between the baseline phase and the task. It then fell sharply again between the task and the post-task phase, reaching levels below those at baseline.

Similarly, in the case of DAP, a decrease was observed during the task, followed by a partial recovery. Finally, FP showed the highest values and a very marked progressive decline throughout the protocol.

For FP, the Friedman test revealed significant differences in alpha-amylase levels across the three measurement time points: χ² (2) = 6.00, p = .050 (exact p = .0498, **Table 4**).

*Post-hoc* comparisons using Wilcoxon tests indicated a decrease in alpha-amylase between the baseline and post-task periods in the FP (Z = −3.28, p = .001), whilst no significant differences were observed between the baseline and task periods (Z = −1.248, p = .212) or between the task and post-task periods (Z = −1.44, p = .147) in this same profile.

In the profiles associated with distress (Z = .500, p = .779) and compulsive use (Z = 2.133, p = .344), the Friedman test showed no significant differences over time.

## Discussion

The aim of this study was to understand the complex interaction between cognitive control, stress regulation and the psychophysiological response to digital social media stimuli across different usage profiles. Through a multidimensional, real-time assessment, we sought to overcome the limitations of traditional self-reports by linking neurocognitive processes (Go/NoGo paradigm) with objective biomarkers such as heart rate variability (SDNN), salivary alpha-amylase and oxytocin, with the aim of characterizing distinct psychophysiological profiles of social media use beyond self-report measures.

The pattern of results observed in the functional profile (FP) supports the validity of the experimental design and the selected biomarkers, given that the functional profile showed the expected response patterns according to previous scientific literature across all analyzed variables, presenting results aligned with health and normality standards.

The functional profile (FP) not only achieved the best cognitive performance, with the highest number of correct responses and the fewest errors, closely aligning with the values reported in the previous literature (43). Descriptively, it also showed the highest sensitivity indices (d’) both overall and in response to social media stimuli; however, these between-group differences did not reach statistical significance (**Table 3**) and should therefore be interpreted with caution rather than as evidence of superior discrimination ability.

Furthermore, it also demonstrated effective autonomic regulation, evidenced by a significant increase in SDNN following the task. The FP exhibited the expected physiological pattern of SDNN decrease during task exposure followed by significant recovery in the post-task phase. This ‘decline and rise’ pattern in SDNN may be indicative of biological health and resilience, potentially reflecting the autonomic nervous system’s ability to adapt flexibly to cognitive challenge and restore homeostasis (74, 75).

Endocrine markers provided partial support for the validity of the measurement approach. Oxytocin showed a non-significant descriptive upward trend in the FP and is not discussed further here. Alpha-amylase showed a progressive and significant decline from baseline to recovery (p = .001), consistent with efficient sympathetic regulation and rapid recovery (53, 76, 77).

Taken together, the consistency of these findings suggests that the variables used are appropriate for distinguishing functional profiles from those that are not, providing preliminary support that should be confirmed in future studies with larger samples.

Turning to the characterization of problematic profiles in social media use, revealing a critical combination of psychological, physiological and behavioral factors. Given the modest subgroup sizes (CUP n = 16, DAP n = 14, FP n = 20), the comparisons discussed below should be interpreted as hypothesis-generating rather than as definitive evidence of distinct psychophysiological profiles.

The CUP and FP profiles exhibited similar responses, with no significant differences in cognitive performance on the inhibitory control test and normally distributed patterns in heart rate results.

In contrast to the other profiles, the CUP showed a descriptive increase in alpha-amylase during task exposure followed by a decrease in the recovery phase. This within-group change did not reach statistical significance (**Table 4**) and should therefore be regarded as an exploratory observation rather than a confirmed physiological mechanism. If replicated in future studies with larger samples, such a pattern could be consistent with heightened sympathetic reactivity to cognitive demand and/or a lower threshold for physiological activation (78), a response profile that has been tentatively associated in the literature with greater impulsivity and lower inhibitory control (79–81), and with high physiological reactivity accompanied by rapid return to baseline (82).

Oxytocin showed a non-significant descriptive rise-and-fall pattern in the CUP; given the absence of statistical significance, this observation is noted only as hypothesis-generating and is not interpreted further.

With regard to the distress-associated profile (DAP), a clear contrast to the other two profiles is observed. It should be noted that the between-group differences reported below, although statistically significant, were close to the conventional threshold (p = .039–.046) and were not corrected for the number of tests conducted; they are therefore reported as preliminary observations requiring independent confirmation.

Firstly, it performed worst in general inhibitory control and in specific inhibitory control in response to social media stimuli, being the only group in our study to show significant differences compared to the healthy control group. This deterioration was characterized by a higher number of omissions both in the task in general and specifically in response to social media stimuli, indicating difficulty in maintaining sustained attention and executing motor responses to relevant cues.

These findings are consistent with recent research in which specific stimuli appear to have a disruptive effect on the cognitive system in vulnerable profiles (43).

Whereas Wegmann et al. (43) reported that attentional deficits manifest primarily as false alarms to social media cues, the present findings suggest a different pattern: a complementary within-subject analysis confirmed that the deficit in inhibitory control in the DAP is specifically associated with social media stimuli: this profile showed significantly more omissions and fewer correct rejections in response to social networking site (SNS) stimuli compared with neutral stimuli (Z = -2.196, p = .028), whereas no such stimulus-specific pattern was observed in the CUP or FP. This finding provides preliminary support for the stimulus-specificity hypothesis and is consistent with models of cue-reactivity in behavioral addictions (83), although it derives from a single within-subject contrast in a small subgroup (n = 14) and warrants replication. This pattern could reflect a state of cognitive overload in response to relevant stimuli, such as a reward bias (84), which compromises both response execution and inhibition (85, 86).

Furthermore, although the TDS-based analysis did not reveal any statistically significant differences, the descriptive findings point to a reduction in discriminative ability, particularly in response to stimuli associated with social media in this profile.

Taken together, these findings suggest that the DAP deficit is not limited to reduced perceptual discrimination ability but rather reflects a response pattern characterized by difficulties in sustained attention and response execution in the face of highly motivational stimuli, likely modulated by these stimuli’s ability to activate states of arousal that interfere with cognitive control.

This cognitive pattern was observed concurrently with an absence of significant SDNN change across phases in the DAP. While reduced short-term SDNN variability has been associated in the literature with lower vagal control (87), SDNN is a global HRV index influenced by multiple physiological and contextual factors, and its stability across phases does not, by itself, demonstrate autonomic dysfunction or an impaired capacity for stress regulation. This finding should therefore be interpreted as a descriptive indicator of autonomic stability rather than direct evidence of regulatory failure.

Regarding salivary biomarkers, although we must exercise caution due to the exploratory nature of the study and the lack of statistical significance, relevant patterns were observed.

Oxytocin showed a non-significant descriptive increase at the end of the task in the DAP and is not interpreted further here.

Descriptively, alpha amylase in the DAP showed a pattern inverse to classical sympathetic reactivity, with a decrease during the task and an increase in the recovery phase. In the absence of statistical significance, this pattern cannot be interpreted as evidence of a specific stress processing mechanism and should be treated as an exploratory observation for future research.

If replicated with statistical significance, this descriptive pattern might be consistent with models proposing misalignments in the timing and coordination between stress response systems in individuals with psychological distress. It is also consistent with those suggesting that greater sAA reactivity has been linked to personality traits such as neuroticism and rumination, exhibiting heightened physiological responses to stress (88).

According to the stimulus–organism–response model with cognitive, adaptive and normative components (SORCAN) proposed by Tan (12), problematic engagement should be understood as the result of dynamic interactions between environmental cues, internal emotional regulation processes, and the individual’s adaptive or normative responses.

In line with this framework, empirical evidence suggests that emotional regulation and maladaptive coping strategies act as mediating mechanisms in the relationship between attentional difficulties and problematic social media use (PSMU) (89).

Consistent with this framework, the present findings extend this approach by highlighting the cyclical nature of this process: the internal state characteristic of the DAP profile, associated with higher levels of distress, could lead to a recoding in the interpretation of external signals (digital stimuli), which, in turn, feeds back into problematic responses.

Taken together, these findings highlight the importance of examining coping mechanisms and regulatory processes as central elements in shaping the networks associated with the PSMU. The vulnerability-stress model supports this theory, suggesting that the negative impact of these networks is greater in individuals who already have a pre-existing vulnerability to mental health problems (90, 91).

In conclusion, this study suggests that problematic social media use does not follow a single pattern but may be mediated by distinct neurophysiological and psychological profiles.

The main contribution of this study appears to be the behavioral and partial autonomic distinction between compulsive social media users and those whose use is associated with distress, a finding that should be considered preliminary given the sample size and the absence of correction for multiple comparisons. Among the physiological measures examined, SDNN provided the most consistent evidence of between-profile differentiation, while mean heart rate, alpha-amylase, and oxytocin showed more limited or non-significant effects. Conclusions regarding biological differentiation should therefore be weighted primarily toward behavioral and SDNN-based findings, with biomarker results from oxytocin and alpha-amylase regarded as exploratory and hypothesis-generating rather than confirmatory. Whilst the CUP showed a descriptive pattern of sympathetic reactivity followed by apparent autonomic recovery, and the DAP exhibited a pattern of autonomic stability across phases, the Scheirer-Ray-Hare test revealed no significant Profile × Phase interaction for any physiological variable, indicating that these descriptive patterns did not differ significantly between profiles and should be interpreted as exploratory observations. Notably, however, a significant main effect of Profile was found for MeanHR (H(2) = 17.584, p <.001), indicating that the DAP exhibited consistently higher mean heart rate levels than the CUP and FP throughout the experiment — the most robust physiological finding of the study. The DAP also showed more omissions in response to social media stimuli, consistent with the stress-tension-outcome (SSO) model; however, given the absence of significant Profile × Phase interactions, physiological interpretations remain exploratory.

Although the present findings are preliminary and require replication before clinical recommendations can be made, the identified profiles may tentatively suggest different intervention priorities. The findings of this study highlight the need to develop tailored interventions for problematic social media use, moving beyond generalist approaches that treat this phenomenon as a homogeneous construct. For the compulsive use profile, interventions should prioritize training in inhibitory control and the modulation of prepotent motor responses, in line with programs based on mindfulness and response inhibition (92). For the profile associated with distress, the therapeutic priority should lie in emotional regulation and stress management. This profile appears to represent a case in which social media may function less as a primary reinforcer and more as a potential chronic stressor; however, the cross-sectional design precludes any causal conclusions regarding the individual’s capacity for physiological regulation (13).

Oxytocin did not show statistically significant changes in any profile; the descriptive trends observed should be treated strictly as hypothesis-generating, and no diagnostic, therapeutic, or mechanistic conclusions can be drawn from the present data.

The interpretation of the results must be considered within the context of several methodological limitations that constrain the scope of the conclusions.

Firstly, the small sample size (N = 50) limits the statistical power to detect small or moderate effects, particularly in variables with high inter-individual variability, such as hormonal and enzymatic responses. Furthermore, the high variability inherent in certain biomarkers means that the results obtained must be interpreted with caution. In particular, the analyses of oxytocin and alpha amylase should be regarded as purely exploratory, given the absence of statistically significant changes within the profile across the phases and the high inter-individual variability inherent in these measurements in small samples. Consequently, studies with larger sample sizes and controlled designs will be necessary before robust conclusions can be drawn regarding the physiological mechanisms involved.

Furthermore, the interpretation of biomarkers requires consideration of certain specific limitations. With regard to autonomic regulation, interpretations are based primarily on the SDNN, a global measure of heart rate variability (HRV) that reflects overall variability but is influenced by multiple physiological and contextual factors and does not directly reflect the capacity for stress regulation. Therefore, caution should be exercised when attributing the absence of significant changes in the SDNN to specific physiological mechanisms, such as rigidity or autonomic dysfunction. With regard to the oxytocin biomarker, it should be noted that the measurement of salivary oxytocin remains a methodologically controversial approach in psychobiological research. Although the monoclonal immunoassay used in this study has been analytically validated in terms of precision and accuracy (71), uncertainty persists regarding the correspondence between peripheral salivary oxytocin levels and central oxytocinergic activity, which limits the interpretation of salivary measurements as direct indicators of central nervous system function.

Another significant limitation is that several confounding variables known to influence HRV, salivary alpha-amylase and oxytocin were not systematically controlled for. Although the assessment sessions were standardized to take place between 12:00 and 18:00 to minimize circadian variability, and participants were instructed to attend on an empty stomach and avoid strenuous physical exercise prior to the tests, variables such as caffeine intake, medication use or the phase of the menstrual cycle were neither assessed nor controlled for. These factors could have introduced additional variability into the biomarker measurements, particularly for oxytocin and alpha amylase; therefore, future research should incorporate them through structured pre-session questionnaires and standardized control protocols. Similarly, the present study did not examine the possible influence of sex as a covariate, despite the existence of well-documented sex differences in both HRV indices and oxytocin reactivity (61, 93). Given that 59.2% of the sample consisted of women, future studies should control for sex as a moderating variable to determine whether the psychophysiological profiles identified are expressed differently between men and women.

From a study design perspective, the main limitation is its cross-sectional nature, which prevents causal inferences from being drawn regarding the direction of the observed relationships. Consequently, no causal conclusions can be drawn regarding the directionality of the associations described throughout the manuscript, including those relating to the stress response, the interpretation of which is necessarily based on assumptions about such directionality. In this context, future research should incorporate longitudinal designs with multiple measurement points to examine whether autonomic rigidity, operationalized in the present study as virtually zero heart rate variability associated with a high heart rate, constitutes a vulnerability factor preceding problematic use, potentially linked to neurotic traits, or whether, on the contrary, it arises as a physiological consequence of prolonged and intensive social media use. Resolving this issue would allow for a better understanding of the etiopathogenic mechanisms underlying social media use disorder and would provide a more solid basis for the development of evidence-based preventive strategies.

With regard to the statistical analysis, whilst the Bonferroni correction was applied to the *post hoc* pairwise comparisons, no correction for multiple comparisons was carried out for the complete set of Kruskal-Wallis tests. This limitation affects not only the comparisons made between cognitive variables, but also, more generally, the high number of statistical tests carried out in the behavioral, autonomic, endocrine and enzymatic domains. Given the number of dependent variables analyzed simultaneously, an increased risk of a Type I error cannot be ruled out. Furthermore, several of the significant findings were very close to the conventional significance threshold (e.g. p = 0.039; p = 0.042; p = 0.046; p = 0.049); therefore, their robustness should be interpreted with caution and confirmed by subsequent studies with larger samples.

Finally, the present study did not include a direct within-subject comparison between social networking service (SNS)-specific stimulus conditions and neutral conditions at the global level. Although a supplementary Wilcoxon analysis confirmed stimulus specificity in the DAP, future research should incorporate explicit contrasts between SNS and neutral stimuli as primary analyses to test the hypothesis of stimulus specificity more rigorously.

## Data Availability

The datasets presented in this article are not readily available because The data supporting the findings of this study are not publicly available due to the sensitive nature of human participant information and the terms of the informed consent under which the data were collected. Requests to access the datasets should be directed to the corresponding authors at myriam.carbonell@ufv.es or e.bernabeu.prof@ufv.es.
