# Supplementary material for: Inhibitory control and stress biomarkers in response to social media stimuli: a Go/No-Go study using heart rate variability, alpha-amylase and oxytocin
Source: Front Psychiatry. 2026 Aug 19;17:1872039. doi: 10.3389/fpsyt.2026.1872039 (PMC13535927; doi:10.3389/fpsyt.2026.1872039)
Supplement: Supplementary file 1 [file SupplementaryFile1.docx]

Supplementary Material

**Table S1**

***Final Cluster Centers from the K-means Cluster Analysis Conducted on the Independent Sample (n = 516)***

| **Variable** | **PUC (*n* = 131)** | **PAM (*n* = 164)** | **PF (*n* = 221)** |
| --- | --- | --- | --- |
| PSNUSS Total | 84.13 | 66.13 | 51.33 |
| Daily Social Media Use (hours) | 3.67 | 3.43 | 2.66 |
| DASS Total | 22.37 | 27.56 | 11.01 |
| OCDUS Total | 58.56 | 47.13 | 36.23 |
| WLEIS Emotional Regulation | 18.14 | 17.51 | 21.01 |

*Note.* Cluster assignment in the current study (n = 51) was performed using the nearest centroid classification method based on Euclidean distance to the final cluster centers reported above. PUC = Compulsive Use Profile; PAM = Distress-Associated Profile; PF = Functional Profile. PSNUSS = Problematic Social Network Use Scale; DASS = Depression Anxiety Stress Scales; OCDUS = Obsessive-Compulsive-related Disorder Use Scale; WLEIS = Wong-Law Emotional Intelligence Scale. Convergence was achieved after 6 iterations (maximum absolute coordinate change = .000; minimum distance between initial centers = 69.788).
