# Supplementary material for: Inhibitory control and stress biomarkers in response to social media stimuli: a Go/No-Go study using heart rate variability, alpha-amylase and oxytocin
Source: Front Psychiatry. 2026 Aug 19;17:1872039. doi: 10.3389/fpsyt.2026.1872039 (PMC13535927; doi:10.3389/fpsyt.2026.1872039)
Supplement: Supplementary file 2 [file SupplementaryFile2.docx]

# Captions for Supplementary Figures and Audio

*Inhibitory control and stress biomarkers in response to social media stimuli: a Go/No-Go study using heart rate variability, alpha-amylase and oxytocin (Frontiers in Psychiatry, Article ID 1872039)*

The captions below describe the 15 supplementary images (on-screen instruction slides from the auditory Go/No-Go task) and the 4 supplementary audio files (the auditory stimuli used in the task), corresponding to Supplementary Images 1–15 and Supplementary Audio 1–4 as listed in the Production Forum.

| **File** | **Caption** |
| --- | --- |
| **Supplementary Image 1** | On-screen instruction slide from the auditory Go/No-Go task: “The first round is about to begin.” Participants are shown the WhatsApp notification icon and a bicycle bell icon (linked by “or”) representing the two tones they must respond to by pressing the spacebar, illustrated with an image of a finger pressing the spacebar. |
| **Supplementary Image 2** | On-screen instruction slide indicating the tones participants should NOT respond to in this block: the Instagram notification icon and a table bell icon (linked by “or”), shown next to an image of a keyboard crossed out in red to indicate no key should be pressed. |
| **Supplementary Image 3** | On-screen instruction slide: “The first round is about to begin.” Participants are shown the Instagram notification icon and a table bell icon (linked by “or”) as the tones to respond to by pressing the spacebar, in the counterbalanced block where the Go stimuli are reversed relative to Image 1. |
| **Supplementary Image 4** | On-screen instruction slide indicating the tones participants should NOT respond to in the counterbalanced block: the WhatsApp notification icon and a bicycle bell icon (linked by “or”), shown next to a keyboard image crossed out in red. |
| **Supplementary Image 5** | On-screen instruction slide introducing the full Go/No-Go task. All four auditory stimulus icons are shown together (WhatsApp, bicycle bell, Instagram, table bell). The text explains that participants must respond by pressing the spacebar to the two designated sounds and withhold response to the other two, that immediate visual feedback (a green checkmark for a correct response, a red cross for an incorrect one) is given after each trial, and that the sound-to-response mapping changes between sessions, so participants must pay close attention to the instructions before each round. |
| **Supplementary Image 6** | On-screen prompt “Press the spacebar to hear the first sound,” shown with the WhatsApp notification icon and an image of a finger pressing the spacebar. |
| **Supplementary Image 7** | On-screen prompt “Press the spacebar to hear the first sound,” shown with the bicycle bell icon and an image of a finger pressing the spacebar. |
| **Supplementary Image 8** | On-screen prompt “Press the spacebar to play the first sound,” shown with the Instagram notification icon and an image of a finger pressing the spacebar. |
| **Supplementary Image 9** | On-screen prompt “Press the spacebar to hear the first sound,” shown with the table bell icon and an image of a finger pressing the spacebar. |
| **Supplementary Image 10** | On-screen instruction slide introducing the practice block: participants are told that a practice task follows so they can become familiar with the procedure, and that they will be told explicitly which sounds to react to just before the task begins. |
| **Supplementary Image 11** | On-screen instruction slide for the practice round: “The first round is about to begin.” Participants are shown the WhatsApp notification icon and a bicycle bell icon as the tones to respond to by pressing the spacebar. |
| **Supplementary Image 12** | On-screen instruction slide for the practice round indicating the tones NOT to respond to: the Instagram notification icon and a table bell icon, shown with a keyboard image crossed out in red. |
| **Supplementary Image 13** | On-screen instruction slide for a second practice round, with the Go/No-Go mapping reversed: participants are shown the Instagram notification icon and a table bell icon as the tones to respond to by pressing the spacebar. |
| **Supplementary Image 14** | On-screen instruction slide for the second practice round indicating the tones NOT to respond to: the WhatsApp notification icon and a bicycle bell icon, shown with a keyboard image crossed out in red. |
| **Supplementary Image 15** | On-screen closing slide for the practice block: “You have just finished the practice rounds. If you have further questions, contact the researcher. Otherwise, you may start the task by pressing the spacebar.” |
| **Supplementary Audio 1** | Auditory stimulus used in the Go/No-Go task: the bicycle bell tone, one of the two neutral/analog cues. |
| **Supplementary Audio 2** | Auditory stimulus used in the Go/No-Go task: the table bell (“bing”) tone, one of the two neutral/analog cues. |
| **Supplementary Audio 3** | Auditory stimulus used in the Go/No-Go task: the WhatsApp notification tone, one of the two social-media-related cues. |
| **Supplementary Audio 4** | Auditory stimulus used in the Go/No-Go task: the Instagram notification tone, one of the two social-media-related cues. |
